# Paracrine Induction of Cardiomyogenic Differentiation in Patient-Specific MSCs Using Conditioned Medium from iPSC-CMs

**DOI:** 10.3390/biomedicines14040919

**Published:** 2026-04-17

**Authors:** Veronika Litvinenko, Rose Alkhateeb, Serafima Romanova, Sandaara Kovalenko, Vitalii Dzhabrailov, Mikhail A. Popov, Mikhail Slotvitsky, Evgeniy G. Agafonov, Vladislav V. Dontsov, Sheida Frolova, Dmitriy I. Zybin, Dmitriy V. Shumakov, Alexander Romanov, Konstantin Agladze, Valeriya A. Tsvelaya

**Affiliations:** 1 Moscow Center for Advanced Studies, 20, Kulakova Str., Moscow 123592, Russia; veronichkall.2003@yandex.ru (V.L.); rosskhati75@gmail.com (R.A.); scherbina_serafima@mail.ru (S.R.); sandaara.romanova@phystech.edu (S.K.); vitalyadd22000022@gmail.com (V.D.); slmxa@mail.ru (M.S.); isheydi02@gmail.com (S.F.); agladze@yahoo.com (K.A.); 2 M. F. Vladimirsky Moscow Regional Research Clinical Institute, Moscow 129110, Russia; popovcardio88@mail.ru (M.A.P.); agafonov.cardiacsurger@mail.ru (E.G.A.); vvdontsov@yandex.ru (V.V.D.); poison1983@inbox.ru (D.I.Z.); sdvtranspl@rambler.ru (D.V.S.); 3 E. Meshalkin National Medical Research Center of the Ministry of Health of the Russian Federation, Novosibirsk 630055, Russia; abromanov@mail.ru

**Keywords:** heart failure, mesenchymal stem cells, iPSC, regenerative medicine, cell therapy, cardiac diseases, cardiomyocytes

## Abstract

**Background/Objectives**: Patient-derived mesenchymal stem cells (MSCs) represent a promising avenue for myocardial regeneration, yet therapeutic application remains limited by inconsistent differentiation capacity and the absence of standardized cardiogenic induction protocols. This study demonstrates a proof-of-concept for guiding patient-specific bone marrow MSCs toward a functional cardiomyocyte phenotype using paracrine signals from differentiating iPSC-derived cardiomyocytes (iPSC-CMs). **Materials and Methods**: MSCs were maintained in conditioned medium from a concurrent, validated iPSC-CM differentiation protocol, with evaluation via immunocytochemistry, optical mapping, and whole-cell patch-clamp recordings. **Results**: Differentiated MSCs acquired organized sarcomeric architecture with cross-striations and displayed spontaneous calcium oscillations with decay kinetics matching source iPSC-CMs (CaT50 ≈ 283 ms vs. 301 ms). In co-culture, MSC-derived cells exhibited synchronized calcium dynamics with iPSC-CMs, confirming functional coupling, while patch-clamp detected hallmark cardiac ion currents (INa, ICa,L, and IKv). Morphologically, MSC-CMs displayed more mature, elongated rod-like shapes. **Conclusions**: Although current densities indicate partial immaturity, their reproducible detection validates successful cardiomyogenic commitment. This “parallel differentiation” platform eliminates donor-specific protocol tuning, providing a streamlined, paracrine-mediated approach to generate autologous cardiomyocyte-like cells for disease modeling, pharmacological testing, and future regenerative applications.

## 1. Introduction

The use of stem cell-based therapies has gained considerable interest due to the regenerative potential of stem cells. In particular, the therapeutic potential of mesenchymal stem cells (MSCs) in cardiac regeneration has become well established due to their multipotency, immunomodulatory properties, and relative availability from sources such as bone marrow (BM-MSCs) [1,2,3,4,5,6,7]. Clinical studies with MSC injection since the early 2000s have demonstrated their ability to improve cardiac function after infarction [8,9,10]. However, a critical obstacle to clinical application is the significant variability in therapeutic outcomes, often attributed to patient-specific factors affecting the efficiency of MSC proliferation and differentiation [11,12,13]. For example, donor age, comorbidities, and immune status may alter MSC functionality [12,14,15], but systematic analyses linking specific clinical parameters to MSC and BM-MSC behavior remain limited.

Increasing evidence indicates that the systemic and local microenvironment critically regulates MSC fate, including proliferation and differentiation [16]. While clinical parameters (e.g., hemogram) reflect the patient’s systemic state and potentially the bone marrow niche health, their direct predictive value for MSC expansion dynamics remains poorly characterized. Crucially, although immune interactions, particularly with lymphocytes, are known to modulate MSC function in vitro and in vivo [17,18,19], their impact on the fundamental proliferative capacity of patient-derived MSCs within a cardiac cohort is underexplored. Similarly, while studies suggest preserved angiogenic potential of MSCs in ischemic conditions [20,21], the direct effect of ischemic comorbidity (e.g., coronary artery disease) on MSC growth kinetics warrants systematic investigation. Despite extensive in vivo studies on cell therapy, the influence of the aforementioned factors cannot yet be fully examined in vitro, as such differentiation processes have not been successfully recreated in cellular models. Therefore, this work focuses on investigating the feasibility and effective differentiation of MSCs in vitro. We also propose protocols for such differentiation designed to closely mimic in vivo conditions.

In parallel, the successful use of MSCs for myocardial regeneration requires overcoming the limited efficiency and directionality of their differentiation into cardiomyocytes in vitro and in vivo. Existing protocols (chemical inducers, genetic engineering) are often complex, insufficiently effective, or unsafe [22]. Epigenetic reprogramming under the influence of microenvironmental factors is considered a promising non-genetic approach to cell fate control [23]. Strategies to enhance MSC differentiation into functional cardiomyocytes, which are critical for structural repair, often rely on synthetic inducers or co-culture systems [24,25,26,27,28,29], which do not fully reproduce the in vivo microenvironment and patient-specific characteristics that affect the success of the differentiation itself and the survival of MSC cells even before differentiation. Thus, strategies that mimic the cardiac microenvironment using biological factors (e.g., conditioned media) appear to be a powerful tool for targeted differentiation of MSCs into functional cardiomyocytes, but they are currently insufficiently reproducible and studied.

The mechanistic rationale for using iPSC-CM-derived conditioned medium is supported by established proteomic and secretome analysis. Robert et al. demonstrated that the cardiac microenvironment during differentiation is enriched with signaling molecules from the Wnt, TGF-β, and BMP pathways, as well as extracellular matrix components such as fibronectin, perlecan (HSPG2), and IGFBP7, which collectively guide lineage commitment [30]. Furthermore, quantitative secretomics by Wolling et al. revealed temporal dynamics of secreted Wnt antagonists (DKK1, DKK4, and CER1), peaking at day 3 of differentiation, suggesting that conditioned medium harvested at later stages carries a mature paracrine signature capable of inducing cardiogenic programs in recipient cells [31].

In this study, we aim to improve the efficacy of autologous MSC therapy for cardiovascular diseases, specifically targeting myocardial damage and fibrosis. To overcome the limited efficiency and directionality of MSC differentiation into cardiomyocytes, we developed an effective protocol for targeted differentiation of patient-specific BM-MSCs in vitro by reprogramming their microenvironment using conditioned medium from iPSC-derived cardiomyocytes (iPSC-CMs). This approach not only mimics the in vivo cardiac microenvironment in an efficient manner but also successfully reprograms BM-MSCs, inducing their targeted differentiation into cells possessing key morphological, phenotypic, and functional properties of mature cardiomyocytes (demonstrating spontaneous calcium oscillations and transverse striation). This opens the way to the creation of patient-specific cardiomyocytes for disease modeling and therapeutic drug screening. The obtained system of initial induction of MSC differentiation into cardiomyocytes using medium from patient-specific healthy iPSC-CM can be further applied as a protocol for the preparation of MSC-based cell therapy.

## 2. Materials and Methods

### 2.1. Obtaining a Bone Marrow Biopsy

Samples were taken from patients at the Moscow Regional Scientific Research Clinical Institute named after M.F. Vladimirsky. Bone marrow tissue samples were obtained from patients during open-heart surgery, immediately after sternotomy, by collecting bone marrow that had already been isolated from the sternum, in amounts of about 1–2 g. The samples were placed in sterile test tubes and immediately processed to isolate BM-MSCs.

### 2.2. MSCs Isolation

Isolation of MSCs from bone tissue began by treating the biopsy with PBS, followed by cutting it into small pieces and incubating it with TrypLE Express (ThermoFisher Scientific, Waltham, MA, USA) for 20 min at 37 °C with 5% CO_2_. TrypLE Express was then inactivated by adding MSC expansion medium, Dulbecco’s Modified Eagle Medium (DMEM) (ThermoFisher Scientific), followed by centrifugation at 1100× *g* for 10 min. The resulting pellet was suspended in DMEM and cultured at a density of 1.5 × 10^5^ per cm^2^. All flasks were coated with 0.1% gelatin (Biolot, St. Petersburg, Russia) for 30 min before seeding the MSCs. The MSC expansion medium was refreshed every three days. Confluence time was defined as the time required to reach over 80% confluency of adherent cells. Images of MSC cultures were taken every three days using an Olympus IX71 fluorescent microscope (Tokyo, Japan).

Bone marrow samples were collected from 18 patients. BM-MSCs were isolated and cultured. The analysis of various clinical parameters and their relationship to the BM-MSC proliferation rate was performed. Clinical data were collected, including age, gender, body mass index (BMI), presence of coronary heart disease, atherosclerosis, and hypertension, as well as concentrations of glucose, lymphocytes, cholesterol, platelets, leukocytes, erythrocytes, neutrophils, and triglycerides. There is a clear relationship between lymphocyte count and the proliferation rate of MSCs (R^2^ = 0.779, *p* = 0.00037). Higher levels are linked to faster growth of MSCs, leading to a quicker confluence. On the other hand, lower lymphocyte levels are associated with a slower MSC proliferation rate, with a marked increase in the time required to reach confluency.

However, the comparison of differentiation and the data presented were obtained from a single patient’s MSCs selected for this study based on proliferation rates. The parameters for all patients can be found in the database: 10.5281/zenodo.16446190.

### 2.3. Immunocytochemistry

A standard protocol for MSC immunostaining was used to study specific MSC markers, including ALCAM (CD166) and Endoglin (CD140). MSCs were dissociated using TrypLE Express, centrifuged at 200× *g* for 7 min, and the resulting pellet was seeded on gelatin-coated glass dishes at a concentration of 50,000 cells per mL. After one week of culturing, the cells were fixed with 4% paraformaldehyde and permeabilized with 0.4% Triton X-100 for 10 min. To stain F-actin, the cells were incubated with Alexa Fluor 488 goat anti-mouse IgG1 (A21121) 1:100 for one hour. The cells were then incubated with a block buffer (1% bovine serum albumin in phosphate-buffered saline, PBS) for one hour at room temperature. Following this, the cells were incubated with primary antibodies overnight at 4 °C, followed by secondary antibodies for 1 h at room temperature. Nuclei were stained with 4′,6-diamidino-2-phenylindole (DAPI). Cells were washed twice with PBS after each incubation step. The cells were analyzed using an inverted fluorescence microscope (Olympus IX71, Japan).

Primary antibodies and working dilutions: Endoglin (PAA980Hu02; CCC, Katy, TX, USA; 1:100); ALCAM (MAA002Hu22; CCC, USA; 1:100); secondary antibodies (Sigma-Aldrich, St. Louis, MO, USA; working dilution, 1:400); Alexa Fluor 594 goat anti-rabbit IgG (H + L) CF^™^ (SAB4600107); Alexa Fluor 647 goat anti-rabbit IgG Atto (40839-1ML-F).

### 2.4. IPSCs Differentiation and Media Collection

In this study, we employed two human induced pluripotent stem cell (hiPSC) lines, m34sk3 and ISMA6L. Professor Zakian’s laboratory kindly provided the iPSC lines used in this study: https://sites.icgbio.ru/zakianlab-ipsccollection/ (accessed on 15 May 2019). Both lines were previously established and fully characterized, and they demonstrated successful cardiac differentiation in our prior work [32]. Cardiomyocyte differentiation was induced using two distinct protocols based on the modulation of Wnt/β-catenin signaling (GiWi strategy) with CHIR99021 [33]. During the differentiation process, the conditioned medium was collected at each medium exchange step and subsequently used for the differentiation of MSCs.

For co-culture experiments, cardiomyocytes derived from the ISMA6L line were seeded as a monolayer. MSCs were pre-labeled with Cyto Tracer Orange 20 μL/mL (Lumiprobe, Moscow, Russia) according to the manufacturer’s protocol and then co-seeded onto the cardiomyocyte layer. This approach enabled the visualization and tracking of MSC localization and morphology within the co-culture system using confocal microscopy.

### 2.5. MSCs Differentiation

MSCs were passaged and seeded onto wells for differentiation into cardiomyocytes using conditioned medium collected from induced pluripotent stem cell (iPSC)-derived cardiomyocytes on day 20 of their differentiation. The differentiation process was initiated by adding an equal volume of this cardiomyocyte-conditioned medium and RPMI 1640 medium (Lonza, Basel, Switzerland) containing B27 Supplement minus insulin (Thermo Fisher Scientific), supplemented with a fibroblast growth factor (FGF) (Sigma Aldrich) and non-essential amino acids (NEAAs) (GIBCO, London, UK). On the following day, 5 μM IWP2 (Sigma Aldrich) was added to the culture. On day 3, the medium was replaced with a 1:1 mixture of RPMI 1640 containing B27 Supplement minus insulin and the day-20 iPSC-derived cardiomyocyte-conditioned medium, supplemented with 10 ng/mL bone morphogenetic protein 4 (BMP4) (Sigma Aldrich) and 10 ng/mL Activin A (Sigma Aldrich). The sequential application of CHIR99021 (Wnt activation), followed by IWP2 (Wnt inhibition), BMP4, and Activin A, recapitulates the biphasic Wnt signaling requirement and TGF-β family modulation established for cardiac specification in pluripotent stem cells [34,35]. This strategy aligns with findings that early Wnt inhibition suppresses anti-cardiac mesoderm genes (e.g., CDX2, MSX1) and promotes efficient cardiomyocyte induction [36]. Two days later, the medium was changed to the iPSC-derived cardiomyocyte-conditioned medium with the addition of 5 μM IWP2. From day 10 onward, the medium was switched to RPMI 1640 containing B27 Supplement with 10% fetal bovine serum (FBS) (Biosera, Cholet, France). Additionally, MSC differentiation was performed in direct co-culture with iPSC-derived cardiomyocytes (iPSC-CMs). For this experimental setup, MSCs were gradually transitioned to an RPMI-based medium: Initially, a 1:1 mixture of DMEM and RPMI 1640 was used, followed by a complete switch to RPMI 1640. Subsequently, 1 mL of fresh medium was replaced every 2 days, and the co-cultures were maintained for a total of 26 days to assess long-term differentiation and integration.

### 2.6. Optical Mapping

Optical mapping of MSC-derived cardiomyocytes was performed using the calcium-sensitive fluorescent dye Fluo-4 AM. Cells were loaded with prewarmed RPMI 1640 medium supplemented with B27 and containing 4 µg/mL Fluo-4 AM at 37 °C for 20 min. Subsequently, the loading solution was replaced with prewarmed Tyrode’s solution (pH 7.4). Calcium-dependent fluorescence was recorded for 30 s using one of two imaging systems: (1) an inverted fluorescence microscope (Olympus IX71) with its accompanying software or (2) an Olympus MVX-10 MacroView fluorescence microscope (Olympus Co., Ltd., Tokyo, Japan) equipped with a high-speed Andor iXon-3 EMCCD camera (Andor Technology Co., Ltd., Belfast, UK). All recordings were analyzed using ImageJ software (v1.54p).

### 2.7. Patch-Clamp Assay

The electrophysiological study was performed on cardiomyocytes differentiated from MSCs. Currents INa, ICa.L, and IKv were recorded at room temperature, 24–25 °C, using the perforated “whole-cell” patch-clamp method. Amphotericin B (Sigma) at a concentration of 0.12 mg/mL was used as a perforating agent. The cardiac cells were placed in a chamber mounted on the slide of an Olympus IX71 inverted microscope. The chamber was perfused with an extracellular solution consisting of 150 mM NaCl, 1.8 mM CaCl2, 5.4 mM KCl, 1 mM MgCl2, 1 mM Na-pyruvate, 15 mM glucose, and 15 mM HEPES (рН = 7.4 NaOH). Patch-clamp pipettes were filled with intracellular solution: 150 mM KCl, 5 mM NaCl, 2 mM CaCl_2_, 5 mM EGTA, 10 mM HEPES, and 5 mM MgATP (рН = 7.2 KОН).

The experiments were carried out using patch clamp installation consisting of the following main elements: digital converter Digidata 1440A (Axon Instruments, Inc., San Jose, CA, USA), amplifier Axopatch 200B (Axon Instruments, Inc., USA), MP-285 micromanipulator (Sutter Instrument, Novato, CA, USA), Olympus IX71 inverted microscope, Humbug noise filter (A-M-Systems, Carlsborg, WA, USA), anti-vibration platform (AVTT75), and temperature controller TC-324C (Warner Instruments, Hamden, CT, USA). For the manufacture of pipettes, the following equipment was used: micropipette puller P-97 (Sutter Instrument), borosilicate glass (BF150-86-10, Sutter Instrument), and microforge (MF-900, Narishige, Tokyo, Japan). Patch-clamp pipettes were made of borosilicate glass with a tip resistance of 2–4 MΩ when placed in the experimental solution. The pipette displacement was corrected to zero just before the formation of GΩ. After the gigaomic contact formation, a quick compensation adjustment of the amplifier instrument compensated for the pipette’s capacitance. Electrical access to the cell during perforation was marked by the appearance of slow capacitive currents that increased in amplitude as pores formed in the membrane with amphotericin.

For recording INa and ICa.L, the following stimulation protocol was used: prestep of −30 mV with a duration of 150 ms and step of 0 mV with a duration of 300 ms. The total IKv current was recorded using a step protocol from −40 mV to +60 mV with a duration of 5 s. And for the I–V curve registration, the protocol was from −30 mV to +60 mV with a step of 15 mV for 2.5 s. AP was recorded using a current-clamp configuration with a 1 nA current stimulus for a duration of 2.5 ms.

The membrane capacity measured using the Clamp 10.2 software ranged from 12 to 30 pF. Clampfit 10.2 (Molecular Devices, San Jose, CA, USA) and Origin Pro 8.1 (Originlab Corporation, Northampton, MA, USA) programs were used for data processing and analysis. The experimental data from the patch-clamp are presented as the dependence of current amplitude, normalized by cell capacity, on membrane potential—average values with a standard deviation. Averaging comes from at least three different cardiomyocytes.

### 2.8. Statistical and Data Analysis

Initial processing of optical mapping and confocal microscopy datasets was carried out in ImageJ (v1.54p). The custom algorithm for activation map generation was developed in Python (v3.11.13) with support from the Matplotlib (v3.10.0), NumPy (v2.0.2), SciPy (v1.15.3), and Pandas (v2.2.2) libraries. The core principle of the algorithm involves enhancing pixel intensity by a defined percentage relative to its mean brightness value. This adaptive thresholding strategy improves detection sensitivity compared to conventional methods that rely on a fixed global threshold applied uniformly across all pixels. Conduction Velocity Estimation: One established approach to functionally characterize intercellular coupling—including gap junction-mediated communication—is to assess the conduction velocity (CV) of excitation propagation across cell clusters. In this study, we derived a conservative lower-bound estimate of CV to benchmark our MSC-derived cardiomyocyte-like cells against the literature’s data for cells with documented Cx43 expression levels. Let L denote the length of a cell cluster, and let T denote the true time required for an excitation wave to traverse this distance. With a frame acquisition interval of Δt = 110 ms, two outcomes are possible: (1) The interval T falls entirely between two consecutive frames, in which case wave propagation across the cluster is captured within a single frame; (2) T spans a frame boundary, yielding an intermediate frame showing partial propagation. The probability of outcome (1) is given by the following:

p=max(0, 1−TΔt)

Using a confidence level of α (e.g., α = 0.05 for 95% confidence) and observing outcome (1) in k consecutive recordings, we obtain the following inequality:

p≥α1k, then T≤Δt(1−α1k)

This yields an upper bound for T and, consequently, a lower bound for CV:

CV=LT>LΔt[1−(1−α)1k]

All quantitative data are presented as mean ± standard deviation (SD). Statistical comparisons between groups were performed using the Mann–Whitney U test (non-parametric), with the significance threshold set at *p* < 0.05. Sample sizes (n) refer to independent biological replicates (distinct differentiation batches from the same MSC line) unless otherwise specified.

## 3. Results

### 3.1. Characterization of Mesenchymal Stem Cells

**Figure 1:**
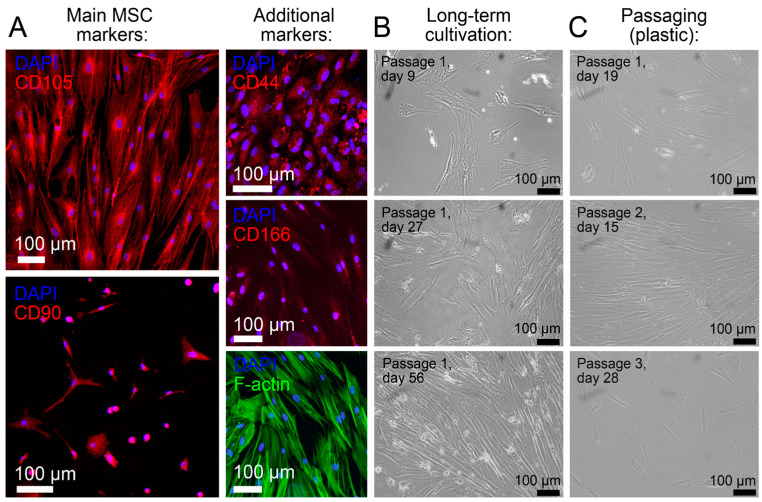
Characterization of bone-marrow-derived mesenchymal stem cells (BM-MSCs). (**A**) Immunofluorescence staining for MSC surface markers. Main markers: CD105 (endoglin) and CD90 (thy-1) show strong membrane-associated expression (red). Additional markers: CD44 and CD166 (ALCAM) also demonstrate characteristic membrane localization (red). F-actin cytoskeleton staining (green) reveals the typical spindle-shaped morphology of MSCs. Nuclei are counterstained with DAPI (blue). Scale bars: 100 μm. (**B**) Phase-contrast microscopy images showing long-term cultivation of MSCs over 56 days. Cells maintain characteristic fibroblast-like morphology and proliferative capacity through extended culture periods (Passage 1, days 9, 27, and 56). Scale bars: 100 μm. (**C**) Phase-contrast images demonstrating MSC morphology during serial passaging on plastic substrate. Cells retain typical spindle-shaped morphology through multiple passages (Passage 1, day 19; Passage 2, day 15; Passage 3, day 28), indicating stable phenotypic characteristics during expansion. Scale bars: 100 μm.

All obtained mesenchymal stem cells (MSCs) were characterized using immunohistochemical markers after isolation from bone marrow. Immunofluorescence staining confirmed the expression of canonical mesenchymal stem cell markers, including CD105 (Endoglin), CD90 (Thy-1), CD44, and CD166 (ALCAM). After one week of culture on adhesion factor-coated glass, cells were fixed and stained. CD105 and CD90 showed strong membrane-associated fluorescence, confirming their identity as main MSC markers (>95% of cells positive by visual assessment in the field of view). Additional markers, CD44 and CD166 (ALCAM), also demonstrated characteristic membrane localization, further validating the mesenchymal phenotype. Staining with Alexa Fluor 488 phalloidin revealed well-organized F-actin filaments and confirmed the typical spindle-shaped, fibroblast-like morphology of MSCs. Nuclei were stained with DAPI (Figure 1A). Long-term cultivation studies demonstrated that MSCs maintained their characteristic morphology and proliferative capacity through extended culture periods (up to 56 days, Passage 1) and multiple serial passages (up to Passage 3), indicating stable phenotypic characteristics during expansion and absence of contamination with other cell types (Figure 1B,C). Phase-contrast microscopy confirmed that cells retained their typical elongated, spindle-shaped morphology throughout passaging on plastic substrate, with no signs of spontaneous differentiation or morphological drift. These findings collectively validate that the isolated cells meet the established criteria for mesenchymal stromal cells and possess the stability required for subsequent differentiation experiments.

### 3.2. MSC Differentiation

After analyzing the data on MSC proliferation, we modeled the process of MSC differentiation into cardiomyocytes after transplantation into the recipient’s heart for cell therapy. Thus, we found a way to induce MSC differentiation into functional cardiomyocytes before transplantation, and we also created a model for studying the behavior of MSCs when used as cell therapy. These two results can be used to improve the efficiency of MSC-based cell therapy. To induce MSC differentiation into cardiomyocytes, we utilized the paracrine signals present in the conditioned medium collected from iPSC-derived cardiomyocytes on day 20. While these iPSC-CMs exhibit a fetal-like phenotype, their secretome contains essential cues to initiate cardiac lineage commitment in MSCs. We have previously demonstrated successful differentiation of human iPSC lines, including those used in this work [32]. These induced cardiomyocyte express factors corresponding to the environment we modeled, thereby inducing MSC differentiation. Such differentiation can check in advance whether the selected MSCs will differentiate into cardiomyocytes and also give an impetus to their differentiation. Transplantation of MSCs already initiated for differentiation is possibly more effective in terms of the absence of arrhythmogenic effects. Thus, pre-conditioning MSCs with iPSC-CM-derived factors provides a promising strategy to prime cells for cardiac repair. This approach could potentially enhance the therapeutic efficacy of MSC-based therapies by ensuring a population of cells already committed to the cardiac lineage before transplantation.

**Figure 2:**
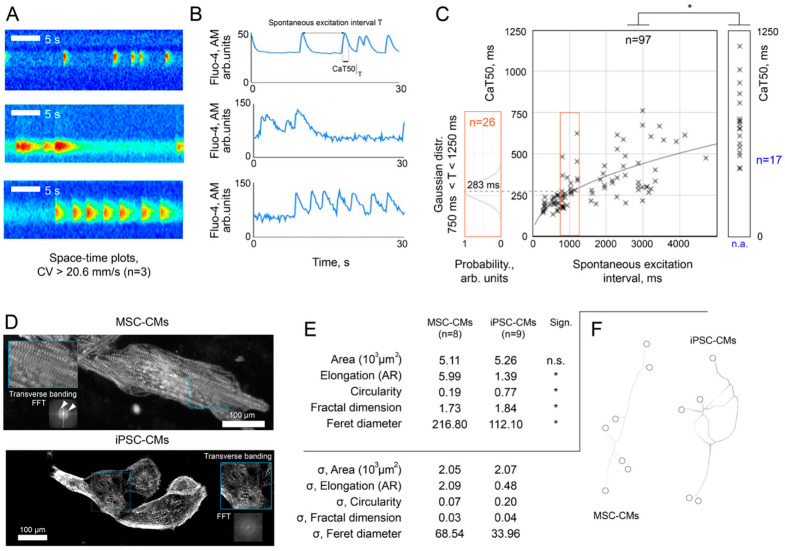
Functional and morphological characterization of MSC-derived cardiomyocytes. (**A**) Space–time plots from optical mapping showing excitation wave propagation in MSC-derived cardiomyocyte clusters. A conservative lower-bound estimate of conduction velocity was derived from n = 19 wave propagation events (frame interval Δt = 110 ms): substituting into the probabilistic model (Section 2.8) yields T < 14.6 ms (95% confidence), corresponding to CV > 20.6 mm/s for typical cluster lengths, indicating functional intercellular coupling. (**B**) Representative calcium transient traces recorded using Fluo-4 AM imaging, showing spontaneous excitation interval (T) and calcium transient duration at 50% decay (CaT50). (**C**) Scatter plot depicting the relationship between CaT50 and spontaneous excitation interval (n = 97 cells). Inset shows the Gaussian distribution of CaT50 values for cells with regular spontaneous activity (750 ms < T < 1250 ms), with a mean CaT50 of 283 ms (n = 26). Cells with irregular activity (spontaneous intervals >5000 ms, representing rare events or first-in-cycle transients) are shown separately (n = 17). Asterisk (*) indicates a statistically significant decrease in CaT50 upon transition to regular spontaneous activity with shorter periods (*p* < 0.01), suggesting functional maturation of calcium handling. (**D**) Immunofluorescence staining for α-actinin showing organized transverse banding patterns in MSC-derived cardiomyocytes (upper panel) and iPSC-derived cardiomyocytes (lower panel). Insets show FFT analysis confirming sarcomeric organization. Scale bars: 100 μm. (**E**) Quantitative morphometric analysis comparing MSC-CMs (n = 8) and iPSC-CMs (n = 9). Upper table shows mean values ± SD for area, elongation (aspect ratio), circularity, fractal dimension, and Feret diameter. Lower table shows standard deviations (σ) for each parameter. Asterisks (*) indicate statistically significant differences (*p* < 0.01); n.s. = not significant. (**F**) Schematic representation of characteristic cell morphology showing more elongated, rod-like shape of MSC-CMs compared to more rounded iPSC-CMs.

The differentiation of MSCs into cardiomyocytes using the differentiation media collected from the parallel differentiation of IPSCs into cardiomyocytes was successfully achieved. Optical mapping of MSC-derived cardiomyocytes using Fluo4-AM showed a clear calcium transient, which indicates the functionality of the cardiomyocytes. Moreover, it showed the fluorescent intensity across multiple cells (Figure 2A,B). Excitation wave propagation analysis across MSC-derived cardiomyocyte clusters yielded a conservative lower-bound estimate of conduction velocity (CV) of > 20.6 mm/s (95% confidence; n = 19 wave propagation events, frame interval Δt = 110 ms; calculation details in Section 2.8). This value falls within the range reported for iPSC-CMs with intact gap junction formation and Cx43 expression [37], providing complementary evidence of functional electrical coupling. We also analyzed the cells obtained during differentiation for cardiac marker, namely, α-actinin. Some of the cells after differentiation demonstrated clear transverse striation of the cytoskeleton, characteristic of contractile cardiomyocytes (Figure 2D). Quantitative assessment of differentiation efficiency revealed that 2.3% ± 1.1% (n = 6 independent differentiation batches) of MSCs exhibited spontaneous calcium transients detectable by optical mapping. By immunocytochemical criteria (α-actinin positivity with clear transverse striation), the efficiency was higher: 7.7% ± 5.0% (n = 6). This discrepancy is expected: Optical mapping detects only cells that are both excitable and functionally coupled to spontaneously active neighbors, whereas marker expression does not guarantee electrophysiological functionality. Both estimates are consistent in order of magnitude with reported efficiencies of directed cardiac differentiation protocols (typically 15–20% when assessed by marker expression alone) [38,39,40,41,42].

### 3.3. Co-Culture of iPSC-CMs and MSC

**Figure 3:**
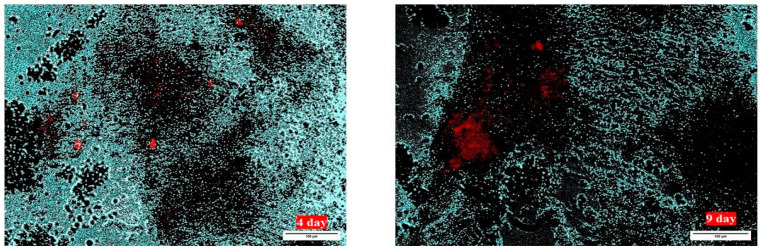
Adhesion and spatial distribution of mesenchymal stromal cells (MSCs) on iPSC-derived cardiomyocyte (iPSC-CM) monolayers. Fluorescence microscopy images are shown for day 4 (**left**) and day 9 (**right**) after seeding of CellTracker Orange-labeled MSCs onto iPSC-CM monolayers. MSCs are visualized in red, while iPSC-CMs appear as dark areas in the background. MSCs demonstrate stable adhesion to the iPSC-CM layer with limited migration over the 9-day observation period, indicating favorable retention characteristics for co-culture applications. Scale bar: 100 µm.

**Figure 4:**
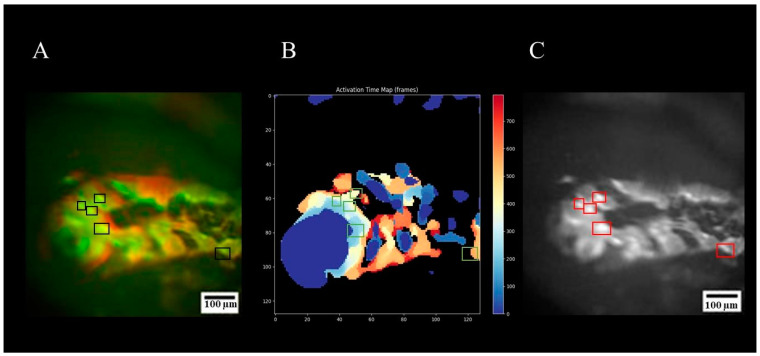
Functional characterization of MSC- iPSC-CMs co-culture. (**A**) Merged image of brightfield microscopy (without filters) and fluorescence microscopy of the co-culture. Colored boxes indicate the locations of MSCs within the co-culture. (**B**) Activation time map of the same region showing the propagation pattern of the excitation wave across the co-culture. The color scale represents activation time in frames. (**C**) Original fluorescence microscopy image showing CellTracker-labeled MSCs (indicated by red boxes) within the co-culture. Scale bars: 100 µm.

To evaluate the interaction between mesenchymal stromal cells (MSCs) and cardiomyocytes, a co-culture model was established using iPSC-derived cardiomyocytes (iPSC-CMs) as a base layer. MSCs were pre-labeled with a fluorescent tracker (CellTracker Orange) and seeded onto the iPSC-CM monolayer. Daily imaging of the co-culture was performed throughout the experiment. The total duration of co-culture was 26 days. Morphological analyses confirmed that MSCs successfully adhered to the iPSC-CM monolayer and maintained stable localization without extensive migration (Figure 3). This adhesion behavior is notable, as MSCs typically exhibit high migratory capacity in vitro, which can complicate their retention in cardiac tissue engineering applications. The stable integration of MSCs within the iPSC-CM layer provided a foundation for subsequent functional assessment of electrical coupling, which was evaluated by optical mapping of synchronized calcium transients (Figure 4).

On day 26, optical mapping of the co-culture was conducted for the functional assessment of the differentiated cells. The recorded calcium transients and generated activation maps demonstrated synchronized contractile activity across the co-culture layer, confirming the functional integration of MSC-derived cells with the underlying iPSC-CM monolayer (Figure 4). This synchronization indicates that MSC-CMs not only adhere to the cardiomyocyte monolayer but also establish functional intercellular coupling following transdifferentiation. While we did not specifically investigate the molecular nature of these contacts, the observed coordinated excitation suggests the formation of functional electrical coupling, which may be mediated by gap junctions (e.g., connexin-based) or alternative mechanisms of intercellular signal transmission (e.g., ephaptic coupling, extracellular field effects, or tunneling nanotubes). Further studies employing immunostaining for specific connexin isoforms (Cx43, Cx40, and Cx41) or pharmacological uncoupling assays would be required to definitively characterize the structural basis of this functional integration.

### 3.4. Patch-Clamp Recording

As part of this study, the electrophysiological activity of cardiomyocytes differentiated from MSCs was studied. The currents of the fast sodium channel INa, the L-type calcium channel ICa,L, and the total current of the potassium channels IKv were recorded.

**Figure 5:**
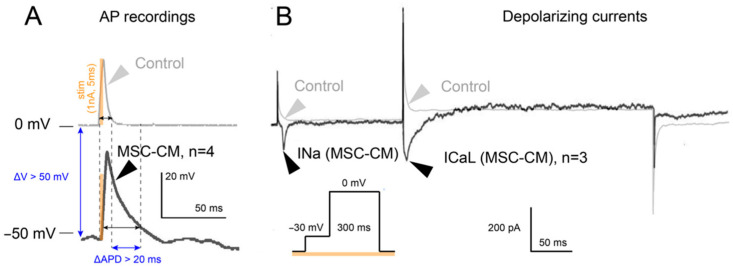
Electrophysiological characterization of MSC-derived cardiomyocytes. (**A**) Action potential (AP) recordings from MSC-derived cardiomyocytes (MSC-CM, black trace, n = 4) compared to undifferentiated MSC control (gray trace). Cells were stimulated with 1 nA current pulses (5 ms duration, orange bar). MSC-CMs exhibit stimulus-evoked action potentials with a characteristic waveform including rapid depolarization and repolarization phases. While absolute resting membrane potential values in stem-cell-derived models may be subject to measurement variability due to membrane fragility and technical factors inherent to manual patch-clamp [43], the key observation is the qualitative emergence of excitable behavior that is absent in undifferentiated MSC controls. Scale bars: 20 mV and 50 ms. (**B**) Representative patch-clamp recordings of depolarizing ionic currents. Fast sodium current (INa) and L-type calcium current (ICa,L) were recorded using a voltage step protocol from −30 mV to 0 mV (300 ms duration, shown in orange). Control traces from undifferentiated MSCs (gray) demonstrate only capacitive artifacts in the absence of functional ionic currents. MSC-CMs show functional INa (black trace) and ICa,L (black trace, n = 3), confirming the presence of key voltage-gated channels characteristic of cardiomyocytes. Scale bars: 200 pA, 50 ms.

**Figure 6:**
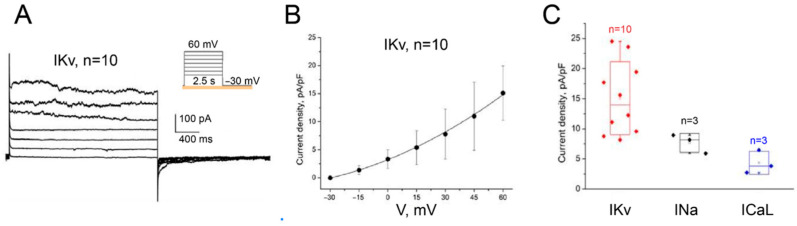
Electrophysiological characterization of MSC-derived cardiomyocytes. (**A**) IKv recorded using an I–V step protocol. (**B**) I–V curve showing voltage-dependent activation of IKv (n = 4 different cells; amplitudes from I–V protocol only). (**C**) Average current densities: IKv = 15.07 ± 6.06 pA/pF (n = 10, amplitudes pooled from i–v and step recordings), INa = 7.66 ± 1.57 pA/pF (n = 3), ICa,L = 4.35 ± 1.92 pA/pF (n = 3). Note: Sodium and calcium currents were more challenging to record and were not consistently detected in all differentiated cells, hence the smaller sample sizes for these parameters.

After the differentiation of MSCs into cardiomyocytes, the general phenotype of the obtained cells was first checked. AP was recorded for these cardiomyocytes. The duration of AP at 80% repolarization (APD80) was 50 ± 4 ms (n = 4). An example of AP recording is shown in Figure 5A. During the process of differentiation of MSCs into cardiomyocytes, calcium, potassium, and sodium currents were also recorded; they are presented in Figure 5B and Figure 6. INa and ICa,L were recorded by the patch-clamp method at room temperature using the step protocol depicted after differentiation (Figure 5B).

Total potassium current IKv in cardiomyocytes obtained by differentiation from MSC. Figure 6A shows the current IKv registered with the I-V protocol (n = 4). By normalizing the amplitude of the currents on the cell capacity, after averaging, the amplitudes of all three currents were obtained in Figure 6. The average current density of INa was 7.66 ± 1.57 pA/pF (n = 3), ICa, L was 4.35 ± 1.92 pA/pF (n = 3), and IKv was 15.07 ± 6.06 pA/pF (n = 10).

## 4. Discussion

The primary outcome of this study is the demonstration of a proof-of-concept for the directed differentiation of patient-specific bone marrow MSCs into functional cardiomyocytes using conditioned medium harvested from differentiating iPSC-CMs. We have shown that this approach successfully mimics the paracrine microenvironment of cardiac tissue in vitro, inducing molecular and electrophysiological changes in MSCs that are characteristic of a cardiomyocyte-like electrophysiology and phenotype.

Stem cell transplantation for cardiac repair continues to attract significant interest. While both embryonic stem cells [44] and iPSCs [45] have been successfully differentiated into cardiomyocytes, understanding the molecular mechanisms underlying their pluripotency and self-renewal is critical for optimizing differentiation protocols [46]. Core transcriptional regulators, such as OCT4, SOX2, and NANOG, form an interconnected network that maintains the undifferentiated state, while signaling pathways including Wnt/β-catenin, TGF-β/Activin/Nodal, and FGF/ERK modulate lineage commitment in a stage-specific manner [46]. Our strategy leverages this knowledge by utilizing the secretome of differentiating iPSC-CMs—which naturally embodies the temporal dynamics of these pathways—to guide MSCs through analogous developmental checkpoints. This paracrine-based approach effectively recapitulates key aspects of cardiac specification without requiring exogenous manipulation of pluripotency factors, offering a physiologically relevant alternative to genetic or chemical reprogramming. MSCs remain a promising autologous cell source due to their accessibility and high proliferative capacity. However, existing protocols for cardiac differentiation—such as those using chemical inducers [24], interleukins [25], microRNAs [47], specialized substrates [27,28], or co-culture with cardiomyocytes [29]—are often technically complex. A major limitation is the need for extensive protocol optimization for each new cell line, a challenge that is compounded by the inherent variability of patient-derived MSCs. Our approach offers an alternative paradigm. Instead of screening for a universal chemical cocktail suitable for diverse MSC lines with variable proliferative potential, we utilize the natural milieu of signaling molecules secreted by cells undergoing a well-established, efficient differentiation protocol. The “signature” of molecules secreted by the iPSC-CMs into the medium acts as an inductive signal, triggering cardiogenic differentiation in MSCs. This shifts the focus from achieving the highest possible efficiency to establishing a universal differentiation method for patient-specific cells, which is particularly valuable when the scale of culture is constrained by the cells’ intrinsic proliferative capacity.

An interesting parallel can be drawn with classical reprogramming techniques. Just as early iPSC colonies were induced by Yamanaka factors and subsequently plated on feeder layers of embryonic fibroblasts, our system uses the conditioned medium from iPSC-CMs as the “reprogramming factor.” The resulting monolayer of iPSC-CMs can then serve as a platform for further maturation of MSC-derived cardiomyocytes (MSC-CMs). This establishes a system of “parallel differentiation”: We first validate an efficient protocol on a control iPSC line and then effectively transfer that success to patient MSCs simply by transferring the conditioned medium. This strategy bypasses the laborious process of protocol optimization for each individual MSC line, a critical advantage when working with cells from elderly patients or those with comorbidities, whose regenerative potential is known to be unpredictable [48,49,50].

The validity of this method is strongly supported by our functional assays. Differentiated cells expressed α-actinin and exhibited cross-striations. Critically, electrophysiological recordings and optical mapping in co-culture provided the most compelling evidence. While the current amplitudes were lower than those seen in mature adult cardiomyocytes—indicating a degree of immaturity typical of in vitro-derived cells and a target for future optimization—their very presence is significant. Furthermore, the ability of these MSC-derived cells to synchronize with iPSC-CMs in co-culture and display coordinated calcium transients demonstrates that even a partially differentiated population can functionally integrate into a tissue sheet. This functional integration reduces the demand for a pure cell population; unlike with iPSC protocols, where undifferentiated cells carry a risk of teratoma formation, any residual MSCs in our system do not pose a similar oncogenic threat and may instead serve a supportive stromal function. This aligns with the concept of MSC secretome-mediated repair, where paracrine factors rather than cell engraftment drive regeneration, offering potential opportunities for cell-free therapy approaches [51]. Recent comprehensive reviews have further emphasized that MSC-derived secretomes can reproduce therapeutic effects comparable to cell transplantation while offering advantages in standardization, safety, and delivery [52]. Critically, secretome-based approaches circumvent challenges inherent to cell-based therapies, including poor engraftment, immune rejection, and arrhythmogenic risk from undifferentiated cells. Our “parallel differentiation” platform extends this paradigm: By demonstrating that the iPSC-CM-derived conditioned medium can actively reprogram MSC fate toward a cardiomyocyte-like phenotype, we provide experimental support for leveraging paracrine cues not only as a supportive adjunct but as a primary driver of lineage commitment. This positions our method as a scalable, cell-free strategy for generating patient-specific cardiac cells for disease modeling, drug screening, or as a priming step prior to autologous transplantation. Regarding the specific mechanistic rationale for the paracrine effect, our approach is consistent with established secretome and proteomic datasets. Le et al. demonstrated that auto/paracrine factors—rather than direct cell–cell contact—are the primary drivers of cardiomyocyte differentiation at low cell density and that early Wnt inhibition via IWP2 can rescue differentiation efficiency by suppressing non-cardiac mesoderm programs [36]. Furthermore, secretome analyses by Robert et al. and quantitative proteomics by Wolling et al. have characterized the extracellular signaling landscape during cardiac differentiation, identifying key mediators in conditioned medium from differentiating iPSC-CMs, including Wnt antagonists (DKK1, DKK4, and CER1), TGF-β family ligands, and ECM proteins that collectively establish a pro-cardiogenic niche [30,31]. While our protocol does not include de novo proteomic profiling of the conditioned medium, the convergence of these established datasets provides a robust mechanistic framework: the conditioned medium likely delivers a temporally coordinated cocktail of morphogens that synergize with the added small molecules (BMP4, Activin A, and IWP2) to guide MSCs through mesodermal specification toward a cardiomyocyte-like fate.

This strategy opens several practical avenues. First, it enables the rapid generation of a minimally sufficient number of patient-specific cardiomyocytes for electrophysiological screening, such as testing for antiarrhythmic drug efficacy, without the need for protracted protocol adaptation. Second, in the context of allogeneic transplantation, this platform allows for a functional comparison. By deriving cardiomyocytes from both donor and recipient MSCs using the same inducing iPSC line, one could assess not only immunological compatibility but also functional electrophysiological compatibility between the donor and the recipient. Furthermore, integrating this differentiation protocol into bioengineering strategies to create 3D cardiac constructs could enhance tissue maturation and structural integration in vivo [53]. The native myocardium is defined by anisotropic architecture, dynamic mechanical loading, and complex cell–matrix interactions that cannot be fully recapitulated in 2D monolayers. Advanced platforms such as decellularized cardiac scaffolds, hydrogel-based bioprinting, and stem-cell-derived cardiac organoids provide physiologically relevant microenvironments that promote sarcomere alignment, electromechanical coupling, and metabolic maturation of cardiomyocytes [53]. Future work should evaluate whether MSC-CMs generated via our paracrine-induction protocol exhibit improved structural organization and contractile function when incorporated into such 3D systems, potentially accelerating their translation toward patient-specific cardiac patches or injectable microtissues for myocardial repair.

This study has several limitations. First, while we did not perform de novo proteomic analysis of the iPSC-CM conditioned medium used in this study, our approach is grounded in established secretome datasets that characterize the extracellular signaling landscape during cardiac differentiation [30,31]. Additionally, we acknowledge that the number of electrophysiological recordings obtained in this study is relatively small. In particular, the sample sizes for sodium (INa, n = 3) and calcium (ICa,L, n = 3) currents are limited, as these currents were technically challenging to record and were not consistently detected in all differentiated cells. While the consistent presence of these currents across successfully patched cells confirms successful lineage induction, these small sample sizes warrant cautious interpretation of the quantitative electrophysiological parameters and highlight the need for future studies with larger datasets to fully characterize the maturation state and ionic profile of MSC-derived cardiomyocytes. Future work will include targeted proteomic validation of the conditioned medium batches used for MSC differentiation to further refine the mechanistic understanding of the paracrine cues driving lineage commitment. Second, our patient cohort did not include young donors (under 30 years of age), which may have masked the well-documented age-related decline in MSC proliferative capacity [48,49,50]. Third, the electrophysiological profile of the derived cells indicates a degree of immaturity. We acknowledge that absolute values of resting membrane potential measured by manual patch-clamp in stem-cell-derived models may be subject to technical variability due to membrane fragility, series resistance artifacts, and seal quality [43]. Notably, the resting potential range observed in our MSC-CMs (−40 to −60 mV) overlaps with values reported for undifferentiated human MSCs [39,40], indicating that absolute potential values alone are not sufficient markers of cardiomyogenic differentiation. Therefore, we emphasize qualitative differences as the primary evidence of successful induction: (1) the emergence of stimulus-evoked action potentials with rapid upstroke and repolarization phases; (2) the appearance of functional voltage-gated INa and ICa,L currents that are absent in undifferentiated controls; (3) the consistency of these findings with independent functional assays (spontaneous calcium transients and organized sarcomeric α-actinin). Achieving full maturation will likely require additional stimuli, such as mechanical loading, electrical stimulation, or prolonged culture. Future studies should also investigate the role of extracellular vesicles (EVs) and mitochondrial dynamics transfer within the conditioned medium, as targeting these pathways may significantly improve metabolic and functional maturity [54]. EVs serve as critical mediators of intercellular communication in the cardiac niche, capable of delivering functional mitochondria, metabolic enzymes, regulatory miRNAs, and pro-survival signals to recipient cells. Given that our differentiation protocol relies on conditioned medium from actively differentiating iPSC-CMs, it is highly probable that EV cargo contributes to the observed paracrine reprogramming and functional integration of MSCs. Proteomic and vesiculomic profiling of our conditioned medium, combined with targeted modulation of EV biogenesis or mitochondrial transfer pathways, could unlock new strategies to enhance the bioenergetic capacity and contractile robustness of MSC-CMs—bridging the gap between fetal-like induction and adult-like functionality. Methodological Considerations Regarding Structural Markers: While we focused on α-actinin with transverse striation as the primary structural criterion for cardiomyogenic differentiation, we acknowledge that comprehensive immunophenotyping—including quantitative assessment of cardiac troponin T (cTnT) and myosin heavy chain (MHC)—represents a valuable direction for future methodological refinement. Our choice of α-actinin striation as the principal endpoint was guided by our previous validation work, in which we demonstrated strong concordance between α-actinin-based morphological assessment and cTnT-positive cell fractions measured by flow cytometry, as well as with functional calcium handling parameters [55]. In the context of this proof-of-concept study, where the primary goal was to establish the feasibility of paracrine-induced differentiation rather than to optimize quantitative efficiency metrics, we considered the convergence of α-actinin striation, spontaneous calcium transients, and electrophysiological activity to be a sufficiently rigorous indicator of successful lineage induction. Future studies employing high-throughput flow cytometry or single-cell RNA sequencing may provide more granular characterization of marker expression heterogeneity within the differentiated population. Fourth, similarly to other studies on MSC-to-cardiomyocyte transdifferentiation, our protocol yields a relatively low differentiation efficiency: 2.3% ± 1.1% (n = 6 independent differentiation batches) by optical mapping criteria (spontaneous calcium transients) and 7.7% ± 5.0% (n = 6) by immunocytochemical criteria (α-actinin positivity with transverse striation). This efficiency is below the “percolation threshold” required for the self-organization of large-scale conducting pathways [56], which currently limits the applicability of this protocol for optical mapping of multicellular clusters—a capability that is critical for drug screening applications [57]. Expanding this protocol for cell therapy and tissue engineering will require a detailed investigation of how intercellular coupling emerges in MSC-derived cardiomyocyte-like cells. Previous studies have shown that functional electrical coupling extends beyond connexin expression profiling [37] and warrants dedicated study across differentiation stages, including the relative contributions of Cx43, Cx40, and Cx41 isoforms. Nevertheless, the consistent registration of sodium, calcium, and potassium currents in the vast majority of patched cells underscores the reproducibility of the differentiation method. In conclusion, our data support the concept that conditioned medium from differentiating iPSC-CMs is a simple and effective tool for inducing cardiogenic differentiation in patient-specific MSCs. This approach standardizes the process of generating cardiomyocytes from diverse MSC lines, mitigates the risks associated with genetic manipulation, and establishes a foundation for an in vitro model of cell-based therapy for myocardial damage, warranting further validation in larger-scale studies.

## 5. Conclusions

This study validates a novel “parallel differentiation” strategy for converting patient-specific bone marrow mesenchymal stem cells (BM-MSCs) into functional cardiomyocyte-like cells using conditioned medium from iPSC-derived cardiomyocytes. We demonstrated that this paracrine-based approach effectively induces organized sarcomeric structures and essential electrophysiological functions, including synchronized calcium handling, without the need for genetic manipulation or complex chemical cocktails. By circumventing the variability inherent in patient-specific MSCs, this protocol offers a potential advantage for improving reproducibility in experimental cell models. Although the differentiated cells exhibit signs of electrophysiological immaturity, their ability to functionally integrate with iPSC-CMs confirms their potential for tissue repair. Notably, comparative analysis revealed that MSC-CMs exhibited calcium transient kinetics comparable to the inducing iPSC-CMs (CaT50 ≈ 283 ms vs. 301 ms) while demonstrating greater structural maturity, as evidenced by more elongated, rod-like morphology. This finding highlights the need for further investigation into the mechanisms underlying this differential maturation pattern, as well as the optimization of cell isolation protocols for improved electrophysiological characterization. Ultimately, this proof-of-concept platform provides a foundation for generating patient-specific cardiomyocyte-like cells, with potential applications in disease modeling and drug screening. Future research will prioritize optimizing maturation conditions and elucidating the paracrine mechanisms driving lineage commitment to enhance the functional potency of these cells for translational applications.

## Data Availability

The raw data announced in this work can be provided upon request: 10.5281/zenodo.19258650 and 10.5281/zenodo.16446190.
